# What you don’t know can’t hurt you: Retro-cues benefit working memory regardless of prior knowledge in long-term memory

**DOI:** 10.3758/s13423-023-02408-w

**Published:** 2023-11-06

**Authors:** Vanessa M. Loaiza, Hiu Wah Cheung, David T. Goldenhaus-Manning

**Affiliations:** 1 Department of Psychology, University of Essex, Colchester, UK; 2 Department of Psychology, University of Sheffield, Sheffield, UK

**Keywords:** Working memory, Long-term memory, Retro-cues, Refreshing

## Abstract

Knowledge stored in long-term memory (LTM) impacts working memory (WM) overall, but it is unclear whether LTM facilitates focusing or switching attention in WM. We addressed this question using the retro-cue paradigm: Briefly presented arrays of individually calibrated numbers of shapes (concrete or abstract) were followed by a blank retention interval (no-cue) or a retro-cue to focus participants’ attention to the to-be-probed shape. Experiment 3 included double retro-cue trials that required participants to switch their attention to a different shape. Participants recalled the color (Experiments 1) or location (Experiment 2) of the probed shape, or recognized the target shape among two other options (Experiment 3). Confirming the overall LTM effect on WM, fewer abstract shapes were needed to match the performance of concrete shapes during the calibration phase. Most importantly, retro-cues benefitted performance regardless of the nature of the shape, suggesting that LTM impacts WM overall without moderating attention.

**Supplementary Information:**

The online version contains supplementary material available at 10.3758/s13423-023-02408-w.

In everyday life, we often must keep a few goals in mind to complete a task, such as following directions to a new restaurant. Working memory (WM) achieves this by keeping a limited amount of information accessible for ongoing cognitive processing (Cowan, 2017). An essential characteristic of WM is to maintain information that is no longer perceptually available. For example, it is not efficient for each step of directions to the restaurant to be continuously repeated to us. Instead, we often think back to the information that was just presented a moment before, but which is currently unavailable in our environment. This process of keeping information active in WM via attentional focusing is called refreshing (Johnson, 1992; see Camos, Johnson, et al., 2018a, for review). The question that we address in this work is whether prior knowledge already stored in long-term memory (LTM) facilitates refreshing in WM. For example, is refreshing more optimal when the restaurant is in our neighborhood compared to a less familiar city? Although prior knowledge in LTM is known to enhance WM overall (Brady et al., 2016; Chung, Brady, et al., 2023a, b; Chung, Tam, et al., 2023c; Engle et al., 1990; Ericsson & Kintsch, 1995; Sobrinho & Souza, 2023), it is more controversial whether refreshing is specifically benefitted. This issue falls against the backdrop of a wider longstanding debate regarding the conceptual overlap of WM and LTM (for recent discussion, see Cowan, 2019; Norris, 2017). Tackling the issue of whether LTM specifically facilitates refreshing in WM thus provides theoretical clarity to the literature by determining one means by which the two memory systems may interact.

One way to investigate the use of attention in WM is the retro-cue paradigm: After a presented array of memoranda (e.g., colors) disappears, a retro-cue guides participants’ attention to the to-be-tested item that is no longer perceptually available (Griffin & Nobre, 2003; Landman et al., 2003; see Souza & Oberauer, 2016 for review). Furthermore, presenting a second retro-cue prompts participants to switch their attention to other memoranda (Chao et al., 2022; Loaiza & Souza, 2018; Rerko & Oberauer, 2013; Rose et al., 2016). Thus, retro-cues indicate which item(s) to refresh in WM, such that relevant information is flexibly swapped in and out of the focus of attention as needed. In both instances of single or double retro-cues that respectively prompt focusing or switching attention, enhanced performance is regularly observed relative to a no-cue/neutral-cue baseline (i.e., a retro-cue effect/benefit), thus evidencing the functional role of attention to augment the accessibility of information in WM.

The current work addresses whether LTM facilitates attention in WM given the considerable recent interest in whether refreshing information into the focus of attention requires retrieving it from a less active state in LTM (LaRocque et al., 2015; Loaiza et al., 2015; McCabe, 2008; Rose et al., 2016). For example, neuroscientific evidence suggests that the neural trace of a representation returns to baseline when it is no longer in the focus of attention (Lewis-Peacock et al., 2012; Rose et al., 2016). So, if bringing less active information into the focus of attention requires its reactivation from outside of WM, then the availability of information in LTM should impact the efficiency of refreshing. The current behavioral literature has shown mixed results, with some work suggesting that LTM moderates the efficiency of refreshing (e.g., Higgins & Johnson, 2009, 2013; Loaiza et al., 2015; Ricker & Cowan, 2010; Shimi & Scerif, 2015), whereas other work suggests that LTM only enhances WM overall without impacting refreshing (e.g., Camos, Mora, et al., 2018b; Labaronne et al., 2023; Loaiza & Camos, 2018). For example, Shimi and Scerif (2015) showed that the retro-cue benefit was stronger for familiar, concrete shapes versus abstract shapes in children and adults. Although this interaction supports the notion that LTM facilitates refreshing, it was an ordinal interaction: The retro-cue effect was smaller but still present for abstract shapes, which showed lower, near-floor performance overall relative to concrete shapes. Our preregistered aim was to ensure that the key interaction between retro-cues and LTM is unambiguously attributable to a facilitatory effect of prior knowledge in LTM rather than an artifact of the scale (Loftus, 1978; Wagenmakers et al., 2012) by using a calibration procedure to achieve a similar baseline level of performance. Overall, the question of whether refreshing involves reactivation from LTM is still unresolved.

A conclusive answer is important not only to understand how WM achieves its essential feature of actively maintaining information, but to also address an enduring debate regarding the relationship between WM and LTM. For example, some models assume that WM and LTM are independent (Baddeley, 2000; Barrouillet & Camos, 2015), thereby suggesting that LTM enhances WM overall without impacting its underlying mechanisms such as refreshing. Other models presume that WM is embedded within LTM (Cowan, 1999; Oberauer, 2002), and thus the efficiency of WM mechanisms like refreshing should be susceptible to what is already stored in LTM. Thus, whether LTM influences refreshing has implications for theoretical conceptions of how WM functions as well as the wider literature regarding the architecture of these memory systems.

In this series of three preregistered experiments,^1^ we addressed this issue more directly than in previous research by varying the prior knowledge of to-be-remembered information presented during a retro-cue paradigm that, as overviewed previously, is widely agreed to vary attention in WM. Participants were briefly presented with arrays of to-be-remembered shapes (either concrete or abstract objects); during Experiments 1 and 2, a retention interval followed that either remained blank (no-cue) or briefly presented the to-be-tested shape in white at the center of the screen (retro-cue). Thereafter, participants recalled the color (Experiments 1) or location (Experiment 2) of the probed shape along a continuous reproduction wheel to assess recall error (i.e., the distance between the true target color/location and the response; see Fig. 1A and 2A, respectively). During Experiment 3, the retention interval either remained blank (0 retro-cues), presented a single spatial retro-cue (i.e., an arrow) pointing to the to-be-tested shape (1 retro-cue), or presented a second spatial retro-cue pointing to a different to-be-tested shape than the first cue (two retro-cues). Participants were informed that the last-presented cue always indicated the to-be-tested shape, and thus they must focus their attention according to the first cue (as in Experiments 1 and 2), but sometimes switch their attention when a second cue is presented. At the end of the trial, participants selected among three possible options presented in one of the colors from the original array: the correct target, a lure that was presented in the trial but in a different color, and a new shape that was not presented in the trial (see Fig. 3A). Thus, all three experiments assessed focused attention with single retro-cues, and Experiment 3 additionally considered switching attention with double retro-cues. Furthermore, Experiment 3 ensured that the pattern of results generalized to spatial retro-cues that required participants to bring to mind the shape themselves and when testing the shape itself using a three-alternative forced choice (3AFC) recognition test rather than an associated feature like color or location as in Experiments 1 and 2, respectively.

**Fig. 1:**
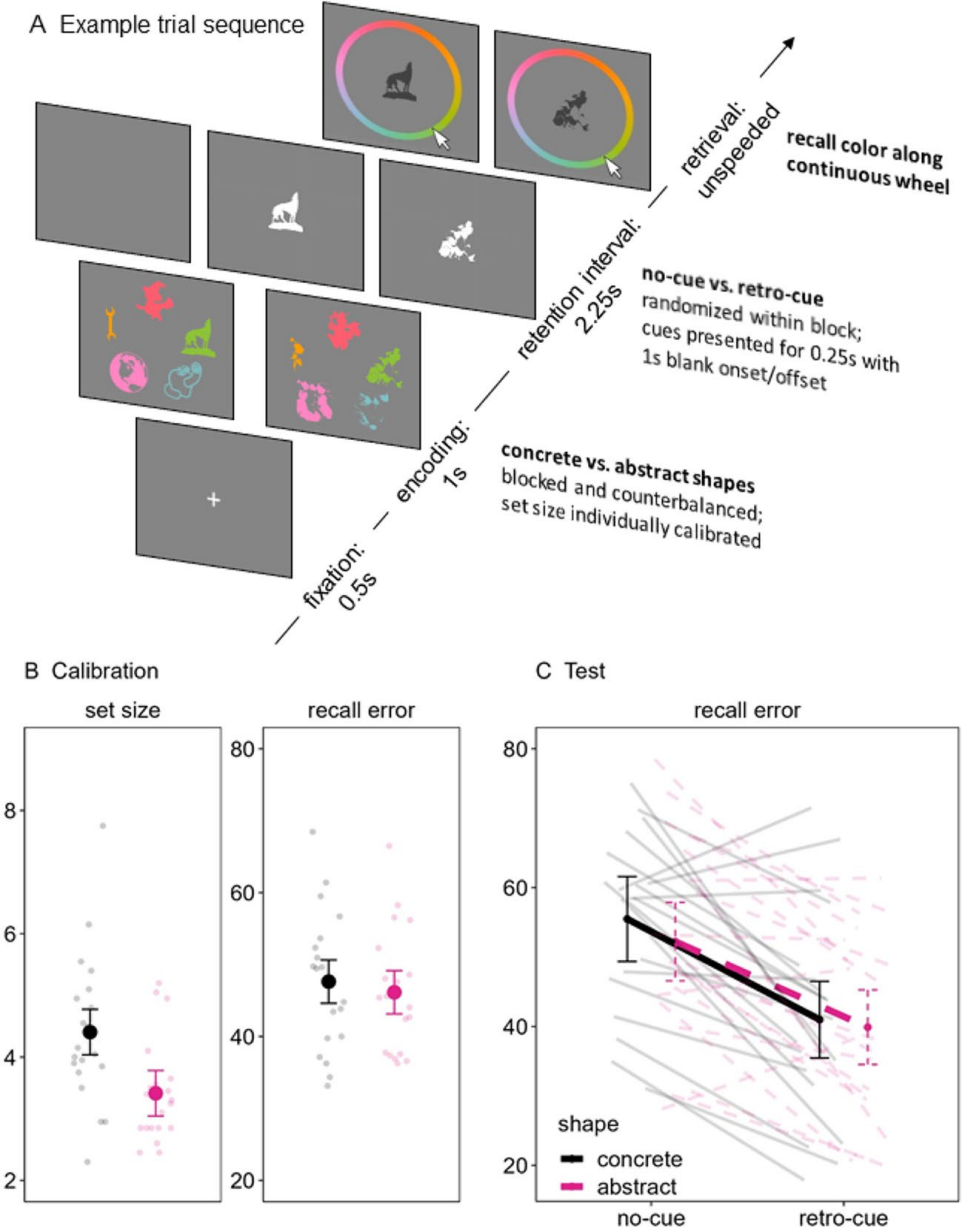
Example trial (**A**) and performance on the calibration (**B**) and test (**C**) phases of Experiment 1. (Color figure online)

**Fig. 2:**
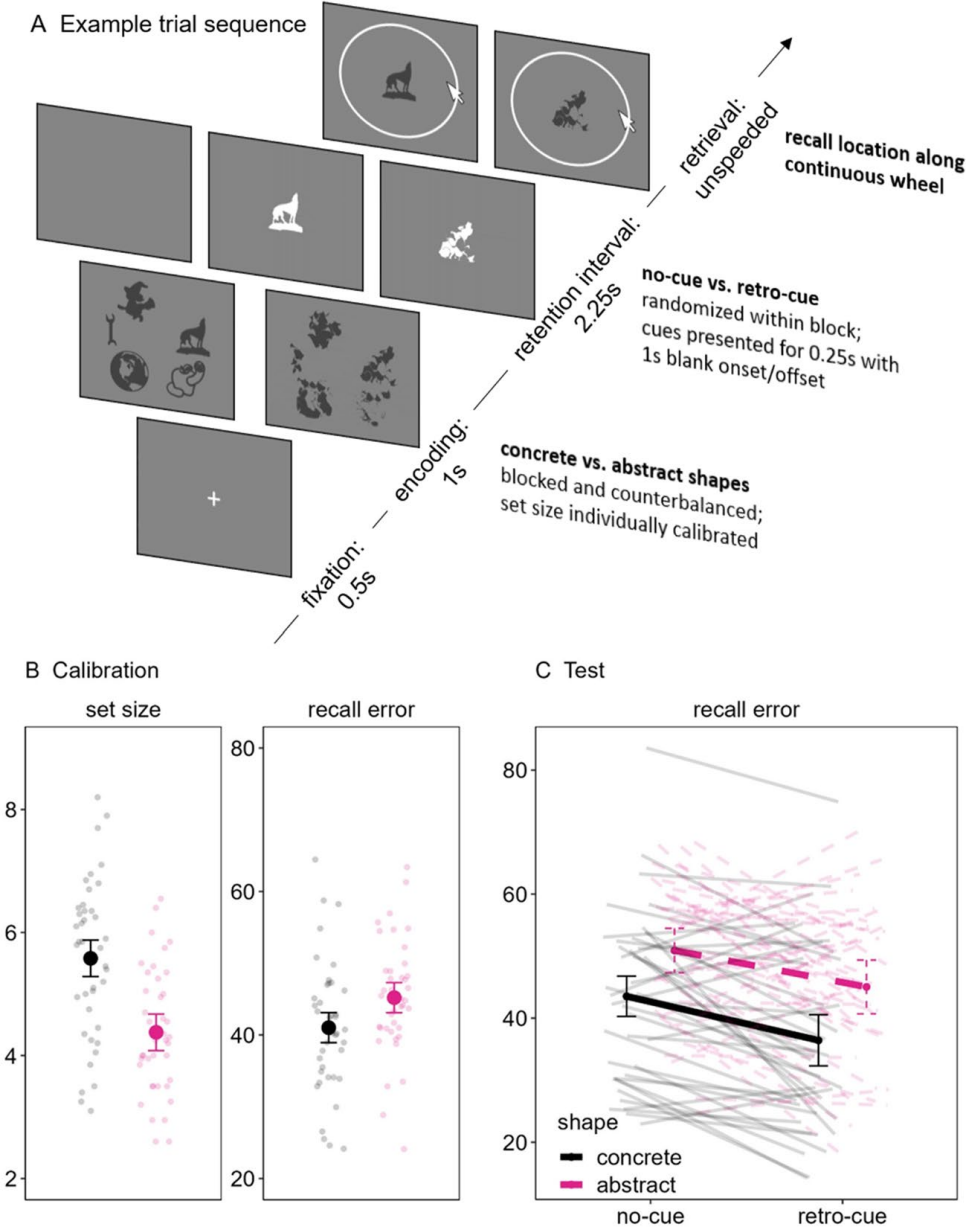
Example trial (**A**) and performance on the calibration (**B**) and test (**C**) phases of Experiment 2. (Color figure online)

**Fig. 3:**
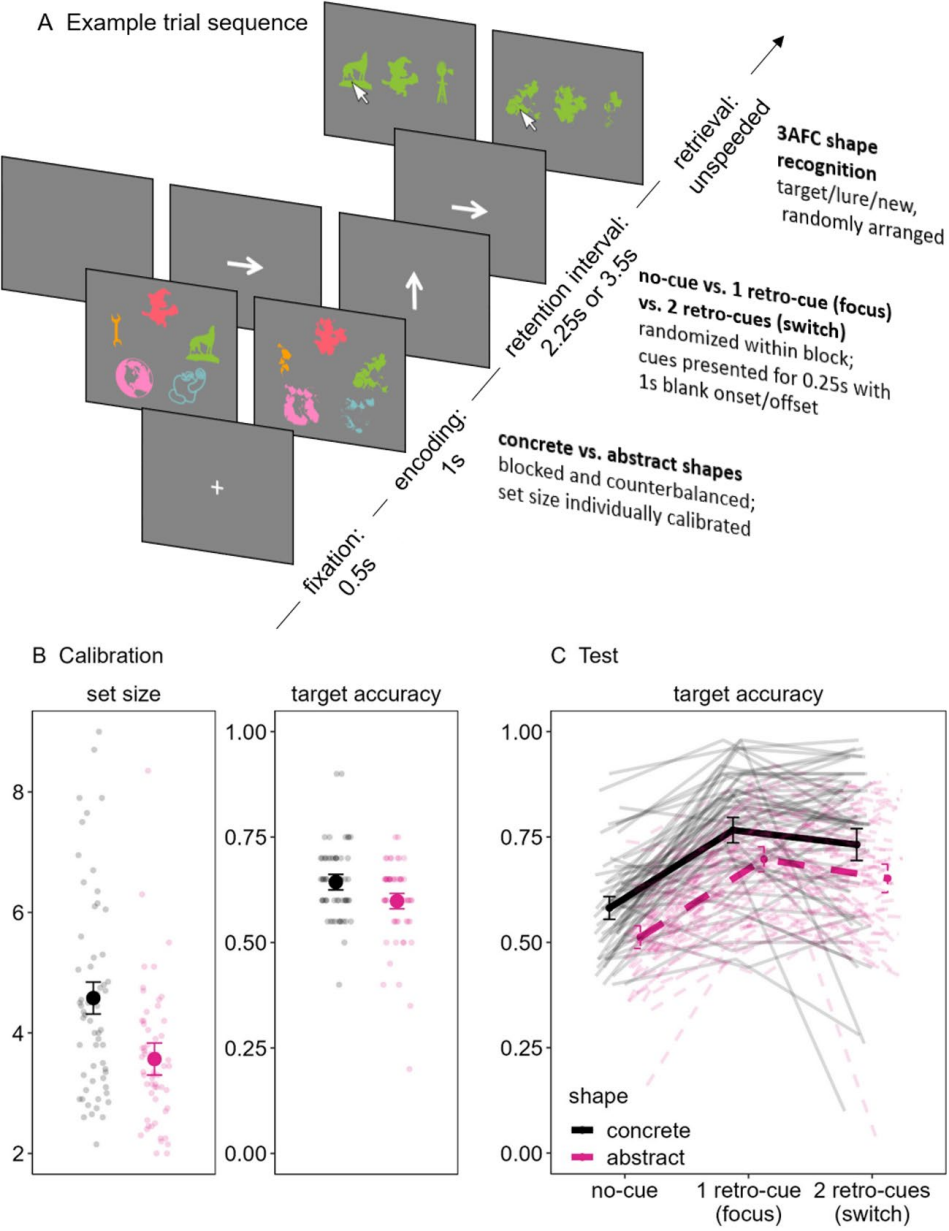
Example trial (**A**) and performance on the calibration (**B**) and test (**C**) phases of Experiment 3. (Color figure online)

To account for the presumed overall benefit of LTM on WM, the task was individually calibrated, such that participants completed two respective no-cue calibration blocks of concrete and abstract shapes to determine the number of each to present during the critical task (e.g., Jaroslawska et al., 2021; Loaiza & Souza, 2019). This ensured that performance was similar between the shapes at the no-cue baseline, thereby allowing unambiguous interpretation of any Shape × Cue interaction (Loftus, 1978; Wagenmakers et al., 2012).

Our hypotheses were as follows: First, if prior knowledge in LTM enhances WM overall, then a fewer number of abstract shapes should be presented to match the performance of concrete shapes during calibration. Having accounted for this difference at the no-cue baseline, we could then address our principal research question regarding the influence of LTM on refreshing in WM: If LTM facilitates refreshing, then the nature of the shapes should moderate the size of the retro-cue benefit, such that retro-cues should more strongly benefit performance of concrete versus abstract shapes relative to a no-cue baseline. Conversely, if LTM does not facilitate refreshing, then retro-cues should benefit performance regardless of the shape relative to a no-cue baseline.

## Method

### Participants

We collected data from UK-based participants who were aged 18–35, native English speakers, with normal color vision (except Experiment 2), and no diagnosed memory or cognitive impairments. Data were considered valid if complete (i.e., the entire experiment was finished) and according to the exclusion criteria explained further on in the Design and Data Analysis subsection. The final sample details are shown in Table 1. Participants were recruited from Prolific (http://www.prolific.co) in exchange for £7.50/hour or the authors’ university subject pool (Sona) for partial course credit. All participants were unique in each experiment (i.e., no participant was allowed to participate more than once or in more than one experiment). All participants were compensated if they finished the experiment without switching windows or restarting it, regardless of the other exclusion criteria explained further on. Participants provided informed consent prior to starting the experiment and were fully debriefed at its conclusion. The University of Essex Ethics Subcommittee 3 approved this project (Protocol Number ETH1920-1706).

**Table 1:** Sample details and exclusions

		Experiment		
Sample details		1	2	3
Total *N* attempted		33	51	120
*N* failed to pass the color blindness/demographic screening phase		6	0	38
*N* excluded for preregistered reasons:		7	11	22
	1. Did not start the experiment after passing the screening phase	3	3	0
	2. Incomplete data (e.g., from exiting the full screen, quitting/restarting)	4	8	20
	3. Reported issues (e.g., technical) affecting performance	0	0	1
	4. Reported not completing experiment in one distraction-free sitting	0	0	0
	5. Recall responses exceeded 5 s on more than 10% of trials	0	0	0
	6. Chance-level recognition performance	–	–	1
Final *N* for analysis after preregistered exclusions		20	40	60

#### Experiments 1 and 2: Focusing attention

We determined a minimum sample size of 20 valid datasets and the number of trials per condition a priori by simulating 150 experiments based on the parameter estimates of a similar prior experiment that used concrete shapes as retro-cues and used a continuous reproduction wheel to test retrieval (Arnicane & Souza, 2021, Experiment 4; see OSF for details). The results of this simulation-based power analysis indicated that 83% of the credibility intervals (CIs) of the retro-cue benefit in the probability of recalling the target (estimate = .114, CI [.046, .182]; see Arnicane & Souza, 2021) did not overlap with zero, thus indicating sufficient power. If initial analyses with this planned sample size did not provide clear evidence regarding our principal hypothesis (i.e., a Bayes factor [BF] greater than 3 for or against the null hypothesis of a similar retro-cue benefit for both concrete and abstract shapes), then we continued sampling in batches of four up to 40 valid datasets. This maximum was also determined from our simulation-based power analysis that suggested that over 99% of the CIs excluded zero with 40 participants.

#### Experiment 3: Focusing and switching attention

We determined a minimum sample size of at least 44 valid datasets a priori by simulating 150 experiments using values (i.e., means and approximate standard deviations) from a similar prior experiment that showed a retro-cue benefit to local recognition of colors using unpredictably presented single and double retro-cues relative to a no-cue baseline (Rerko & Oberauer, 2013, Experiment 1A). The OSF contains the results of the power analysis, but to summarize, the combination of effect/sample size parameters and results for which we intended to power suggested that this minimum would be sufficient to determine no difference (i.e., Cohen’s *d* = 0) or a medium to large difference (i.e., *d* ≥ 0.4) in the double retro-cue effect between shape conditions. However, we planned that up to 60 participants may be required to detect a more subtle difference between shape conditions (*d* = 0.2).

### Materials

The stimuli and the materials to make them, as well as the open-source scripts for all the experiments, are available on the OSF, and a short example can be tried at this link: https://bit.ly/RefLTMexp4. All the experiments were conducted online via lab.js (Henninger et al., 2021) and hosted on the JATOS server Mindprobe (https://jatos.mindprobe.eu/; Lange et al., 2015).

A pool of 504 concrete shapes was adapted from the nameable images developed by Sutterer and Awh (2016) and Sobrinho and Souza (2021). A corresponding pool of 504 abstract shapes was created based on the same nameable images via a diffeomorphic transformation that scrambled the images, thereby resulting in semantically meaningless shapes that still retained the perceptual properties of the original images (Brady & Störmer, 2022; Stojanoski & Cusack, 2014). The pilot experiment confirmed that these scrambled shapes were nearly impossible to identify (see SOM). For each participant, half of the set of 504 shapes was randomly selected to serve as the concrete shapes, while the other half served as the abstract shapes for the whole experiment. The stimuli for each trial were randomly selected without replacement: the shapes from either set (concrete or abstract), and the colors^2^ of the shapes (Experiments 1 and 3) or the locations^3^ of the shapes (Experiment 2). During the trials, the shapes (on-screen size = 90 pixels) were displayed equidistantly around an invisible circle (radius = 150 pixels) in Experiments 1 and 3; in Experiment 2, the locations of the shapes were randomly determined around the invisible circle.

### Procedure and design

Participants first completed a brief demographics survey followed by a color blindness test (except Experiment 2) to check eligibility according to the previously explained inclusion criteria. Participants who did not pass the screening were returned to Prolific/Sona, whereas those who passed were invited to continue onto the main experiment. Prior to starting, the experiment task filled the screen, and participants were informed both in the study advertisement and during the experiment that any of the following actions could risk their data being excluded and uncompensated: Failing to complete the experiment in one continuous, distraction-free sitting; reloading the page; hitting the back button; quitting and restarting the study; escaping the full screen mode/switching windows; or failing to respond to more than 10% of the trials. If participants switched windows away from or exited the full screen, the experiment immediately quit, whereas the other exclusion criteria were implemented during the data pre-processing (see Analytic Procedure).

The experiments were very similar; their differences are explained further on.^4^ All the experiments comprised four blocks of a visual WM task, wherein a calibration block was immediately followed by a test block of either concrete or abstract shapes, with the order of the shape condition counterbalanced across participants (e.g., calibration-concrete, test-concrete; calibration-abstract, test-abstract). Participants received instructions and four practice trials before starting each block, and the experiment concluded with a final survey enquiring about the participants’ experience with the task. The entire experiment lasted about 35–45 min (Experiments 1 and 2) or 50–60 min (Experiment 3) for most participants.

#### Experiments 1 and 2: Focusing attention

Figures 1A and 2A, respectively, show examples of a trial sequence from Experiments 1 and 2. The main difference between them was that participants recalled the color (Experiment 1) or location (Experiment 2) of the probed shape.

Each trial began by displaying a fixation cross for 0.5 s followed by an array of shapes (either concrete or abstract, depending on the block) for 1 s. After a retention interval of 2.25 s, participants were presented with one of the shapes in dark grey at the center of the screen, probing them to recall its color/location by using the mouse to click along a continuous

reproduction wheel. As the participants moved the mouse around the wheel, the color/location of the shape adjusted accordingly. Participants had unlimited time to decide, but they were encouraged to respond as quickly and accurately as possible. Recall error (i.e., the distance between the target color/location and the participant’s response) was recorded. After responding, the next trial began after an intertrial interval of 1 s.

During the calibration blocks, the set size of the array of each trial was adjusted according to the participants’ ongoing recall error across 40 trials. The initial set size of the first four trials of the calibration blocks was five shapes. The set size of the subsequent calibration trials decreased or increased by 1 if participants’ average ongoing recall error of a moving window of the last four trials exceeded or fell below 40°, respectively. There was a minimum of two and a maximum of nine shapes. The average set size in the last 20 calibration trials determined the set size of the subsequent test block. For example, if the average set size was 5.3 concrete shapes, then the subsequent test block contained a memory array with five concrete shapes for 70% of the trials and six concrete shapes for 30% of the trials (see Loaiza & Souza, 2019, for a similar approach).

During the test blocks, the trials were very similar to those of the calibration blocks except that the retention interval of half of the trials either remained blank for 2.25 s (no-cue) or presented one of the shapes to indicate which would be tested with 100% validity (retro-cue). This means that there were no retro-cue trials in which a non-cued item was tested. The shape retro-cue was presented in white at the center of the screen for 0.25 s, with a blank onset of 1 s and a blank offset of 1 s. There were 50 trials of each cue condition, randomly intermixed within the concrete and abstract shape test blocks (i.e., 100 trials in each test block, 200 total test trials in the experiment). Participants had an opportunity for a break and received feedback (both in terms of their mean recall error and expressed as a percentage; i.e., 100 – 100 * mean error/180) after the practice trials and every 10 test trials during both the calibration and test blocks. Furthermore, the duration of participants’ retrieval responses was checked after every trial, and participants received a warning during the breaks about the number of slow-response trials (i.e., trials exceeding 5 s) in the last 10 trials along with a reminder of the instructions to ensure that their attention was focused on the task.

Both experiments followed a 2 (shape: concrete, abstract) × 2 (cue: no-cue, retro-cue) within-subjects design, with the first factor blocked and counterbalanced across participants and the second factor randomized within each block. The dependent variable is recall error.

#### Experiment 3: Focusing and switching attention

Figure 3A shows an example of the trial sequence of Experiment 3. Experiment 3 was very similar to Experiment 1, except that we used spatial retro-cues and included trials with two successive retro-cues to prompt participants to focus and then switch their attention in WM. Furthermore, we tested memory for the shape itself rather than an associated feature using a 3AFC recognition test.

Just as in the previous experiments, the calibration phase entailed trials wherein the set size of the colored shapes (presented for 1 s followed by a 2.25 s retention interval) adjusted based on each individual participant’s ongoing performance, with an average set size of the last 20 trials determining the set size of the subsequent test phase trials for that block. The aim was to achieve a similar level of performance between the concrete and abstract blocks (i.e., approximately 60%–65% target accuracy. A short pilot of the calibration phase (*N* = 18; see OSF) showed that a moving window of an average of the last three trials (rather than four trials, as in the previous experiments) was sufficient to achieve similar performance in this range between the concrete and abstract blocks.

During the test phase, participants viewed a random mix of no-cue, single-cue, and double-cue trials (50 trials of each cue condition). In these test trials, the retention interval lasted 2.25 s (zero or one cues) or 3.5 s (two cues) following similar prior work that intermixed single and double retro-cues (Loaiza & Souza, 2018; Rerko & Oberauer, 2013). The retention interval remained blank during the no-cue trials. The retro-cue trials presented spatial cues (i.e., arrows pointing to the to-be-probed shape) for 0.25 s following 1 s offset (one and two cues) and 2.25 s (two cues) of the memory array. The participants were informed that the last-presented cue indicated with 100% validity which of the shapes would be tested, and so the double retro-cue trials required participants to switch their attention to another shape in WM.

All the trials of both calibration and test phases ended with the participant making an unspeeded decision to select among three options presented in one of the original colors from the memory array: the correct target, a lure that was presented during the trial but in a different color, and a new shape to the trial. During the test trials, approximately half of the lures were randomly determined to be spatial neighbors for trials with set sizes greater than 4; for set sizes of 2–3, the lures are necessarily always spatial neighbors. Furthermore, for double retro-cue trials, approximately half of the lures were randomly determined to be previously cued shapes for set sizes greater than 2; for set sizes of 2, the lures are necessarily always previously cued. This follows similar previous work (Rerko & Oberauer, 2013) and allowed for greater investigation into potential differences between shape conditions in lure selection (see SOM).

Experiment 3 thus followed a 2 (shape: concrete, abstract) × 3 (cue: no-cue, 1 retro-cue, 2 retro-cues) within-subjects design, with the first factor blocked and counterbalanced across participants and the second factor randomized within each block. The primary dependent variable is target accuracy. However, we also considered differences in lure selection during the test block according to whether the lures were spatial neighbors (for all cue conditions) or previously cued (for the double retro-cue condition) for both shape types. To foreshadow, whether the lures were spatial neighbors or previously cued had no impact on lure selection, and so for the sake of brevity we report these results in the SOM and OSF.

### Analytic procedure

We anonymized the data by stripping the Prolific/Sona IDs and replacing them with random ID numbers before depositing the raw data on the OSF. The analysis scripts to reproduce the analyses and figures from start to finish are also available on the OSF.

All practice trials were excluded from analysis. Incomplete datasets were removed from analysis. Data were excluded and replaced for the following reasons: Participants who noted that they experienced legitimate technical difficulties (e.g., internet disruption) that impacted their performance; participants who noted that they did not complete the experiment in one continuous, distraction-free sitting; and/or participants whose responses exceeded 5 s on more than 10% of the trials. In Experiment 3, participants whose overall accuracy performance during the calibration and/or test phases had approached or fell below chance level (i.e., less than 40%) were also excluded. In fact, most exclusions occurred because participants either did not start or failed to complete the experiment (see Table 1).

We used the R package BayesFactor (Morey & Rouder, 2015) with its default settings to assess observed performance (recall error in Experiments 1 and 2; target accuracy in Experiment 3). Specifically, we compared the likelihood of one model (e.g., a model assuming a similar retro-cue benefit for both concrete and abstract shapes, M_cue_) to that of another (e.g., a model assuming a greater retro-cue benefit for concrete versus abstract shapes, M_interaction_) given the data. The ratio of these models is the BF, which is used to update prior beliefs about the relative evidence between two models (e.g., BF_interaction/cue_). BFs are interpreted continuously: BFs between 1 to 3 (or 1 to 0.3) are considered ambiguous, whereas BFs greater than 3 and 10 (or less than 0.3 and 0.1) are substantial and strong evidence for the model in the numerator (or denominator) of the ratio, respectively (Jeffreys, 1961). For example, to draw inferences for our principal hypothesis regarding the interaction between prior knowledge and the retro-cue effect, we checked whether the best model that either included or omitted an interaction was greater than the next-best model by a BF of 3. We ensured reliable estimates of BFs by conducting the analyses with 100,000 iterations.

Furthermore, we fit recall error of Experiments 1 and 2 with a hierarchical Bayesian three-parameter mixture model (Oberauer et al., 2017) and frequency of recognition responses of Experiment 3 with a hierarchical Bayesian multinomial processing tree model (Heck et al., 2018). We also analyzed response times during retrieval across all the experiments. We report these analyses in the SOM and on the OSF, as they were not crucial to the preregistered hypotheses nor did their results provide substantially greater insights than the results reported here.

## Results

### Calibration phase: Does prior knowledge in LTM enhance WM overall?

To address our first preregistered hypothesis, we used a one-sided Bayesian *t* test to compare the set sizes determined during the two calibration blocks of concrete and abstract shapes. We expected that the set size to achieve similar performance between blocks would be larger for concrete versus abstract shapes, thus supporting previous work that prior knowledge in LTM enhances WM performance overall. Although less central to the focus of this project, this was still an important benchmark to establish that LTM enhances WM overall and to ensure that performance was similar at the no-cue baseline to allow for unambiguous interpretation of any interaction during the test blocks.

Experiment 1 showed very strong evidence for the predicted difference in set size between concrete and abstract shapes (H_1_) relative to the null model (H_0_; BF_10_ = 21.27; see Fig. 1B). Experiment 2 replicated and extended these findings by testing a different feature of location rather than color, again showing that the calibrated set size for concrete shapes was overwhelmingly greater than that of abstract shapes (BF_10_ = 1500.88; see Fig. 2B). This was also the case when testing memory for the shape itself rather than an associated feature in Experiment 3 (BF_10_ = 440.32; see Fig. 3B). Note that these differences in Experiments 2 and 3 were likely underestimated given that performance was not completely matched between the shape conditions as we had intended. That is, performance was still slightly better for concrete versus abstract shapes (Experiment 2: BF_10_ = 1.89; Experiment 3: BF_10_ = 3.82) despite the calibration procedure being effective to match performance between the shape types in Experiment 1 (BF_01_ = 3.67). We will return to this point in the next section.

### Test phase: Does prior knowledge facilitate refreshing in WM?

Second and most importantly, we assessed the evidence for our principal hypothesis regarding whether LTM facilitates refreshing in WM by using a 2 (shape) × 2 (cue) repeated-measures Bayesian analysis of variance (BANOVA) on performance during the test blocks. If refreshing in WM at least partially relies on LTM, then the best model should include an interaction between shape and cue, such that the retro-cue benefit is evident for concrete shapes but is either weaker or null for abstract shapes relative to a no-cue baseline. Conversely, if refreshing is independent of LTM, then the best model should omit the interaction, such that there is a similarly strong retro-cue benefit for both concrete and abstract shapes relative to a no-cue baseline.

The design of all three experiments allowed us to test this hypothesis when participants *focus* their attention with a single retro-cue via a 2 (shape: concrete, abstract) × 2 (cue: 0, 1) BANOVA. In Experiment 1, there was overwhelming evidence for an overall retro-cue benefit (BF_10_ = 5362.63) that did not interact with the type of shape (BF_cue/interaction_ = 9.84; see Fig. 1C). In Experiment 2, the best model included main effects of both shape and cue (BF_10_ = 15,202.31; see Fig. 2C), but this was likely due to the previously noted issue in which there was still a slight difference in performance between the concrete and abstract shapes at the calibration phase. Notwithstanding, the main effects model was still substantially preferred to the model including an interaction (BF_main effects/interaction_ = 4.19). Similarly, Experiment 3 also showed overwhelming evidence for a model including both main effects (BF_10_ = 2.13e+29) and substantial evidence against an interaction (BF_main effects/interaction_ = 5.25). Overall, these results indicate that the prior knowledge of the shape did not moderate the single retro-cue benefit, thus providing evidence that LTM does not moderate the efficacy of focusing attention in WM.

Experiment 3 further allowed us to test the hypothesis that prior knowledge may impact refreshing when *switching* attention during double retro-cue trials. A 2 (shape: concrete, abstract) × 2 (cue: 0, 2) BANOVA revealed overwhelming evidence in favor of two main effects (BF_10_ = 2.28e+16) and substantial evidence against an interaction (BF_main effects/interaction_ = 4.92).^5^ Thus, similar to the previous results, the nature of the shape did not moderate the double retro-cue effect, indicating that prior knowledge in LTM does not moderate switching attention to other presumably deactivated information in WM.

## Discussion

Does what we already know impact attending to information that is actively held in mind? The current experiments addressed this question more directly than previous research by varying the extent to which to-be-remembered information is represented in LTM (concrete versus abstract shapes) during a visual WM task that manipulated attention to that information (no-cue versus retro-cues).

In line with our first preregistered prediction and an otherwise uncontroversial view in the literature, our results showed an overall effect of LTM on retrieval from WM, such that a greater number of concrete shapes were needed to calibrate performance to match that of the abstract shapes in all three experiments. This occurred regardless of whether participants retrieved an associated feature of the shape (color or location; Experiments 1 and 2, respectively) or the shape itself (Experiment 3). This provides a novel extension to the literature by showing the overall benefit of prior knowledge in LTM on the calibrated set size rather than performance. Besides a novel approach of evidencing the impact of LTM on WM, the calibration procedure was instrumental to the most important aim of the experiments to investigate whether the nature of the shape moderated the retro-cue effect in the test phase when accounting for this LTM benefit at a no-cue baseline. In all three experiments, we observed consistent evidence *against* a Shape × Cue interaction, such that focusing and switching attention via retro-cues benefitted retrieval from WM regardless of the shape’s representation in LTM. Overall, these findings greatly clarify two outstanding issues regarding how refreshing functions and whether WM and LTM should be considered dissociable memory systems.

First, the consistent retro-cue effect for both concrete and abstract shapes suggests that focusing and switching attention functions similarly regardless of whether the to-be-refreshed information is represented in LTM. This clarifies the mixed results of prior work regarding whether refreshing relies on reactivation from LTM. Here, we have more directly addressed this issue using the retro-cue paradigm that is widely agreed to vary the use of attention in WM.^6^ Experiments 1 and 2 showed similar single retro-cue effects for both concrete and abstract shapes, suggesting that LTM does not moderate focused attention in WM. One could argue that refreshing does not only entail focusing attention, but also includes other aspects such as switching attention to other relevant information in WM (Loaiza & Souza, 2018) and preserving focused attention after distraction (Loaiza & Souza, 2019). This is highly relevant to the current work given the notion that switching attention in WM may rely on LTM (e.g., Rose et al., 2016). Thus, it may be the case that focusing attention in WM (e.g., a single retro-cue pointing to a just-perceived memory item) does not rely on LTM, but LTM may become influential to refreshing if that information must be switched back into the focus of attention. However, Experiment 3 showed that this was not the case: Similar double retro-cue effects were also shown for concrete and abstract shapes, suggesting that focusing and switching attention both function effectively regardless of the availability of information in LTM. Furthermore, the results are congruent with a growing literature showing similar retro-cue benefits regardless of the type of cue (e.g., shape cues in Experiments 1 and 2 vs. spatial cues in Experiment 3; Arnicane & Souza, 2021; Goldenhaus-Manning et al., 2023).

These results have implications for recent work investigating whether reactivating information into the focus of attention involves LTM (e.g., Camos, Mora, et al., 2018b; Chao et al., 2022; Loaiza et al., 2015; Rose et al., 2016). As overviewed in the introduction, there is growing interest in whether representations outside of the focus of attention in WM require reactivation from LTM. The current project shares the logic of some of this prior work that manipulating the status of the memoranda in LTM (e.g., words versus nonwords, low- versus high-frequency words) should reveal whether refreshing at least partially relies on reactivating information from LTM. However, a great deal of this prior work is limited by the fact that the manipulations of refreshing are often indirect (e.g., manipulating the cognitive load of distractors) and that there is often already an effect of LTM at baseline, resulting in ordinal interactions that are ambiguous to interpret. The novel methods of the current experiments extend beyond these limitations, thereby allowing a more unequivocal conclusion that refreshing in WM does not rely on LTM. This conclusion coheres with other recent work suggesting that items outside the focus of attention are unlikely to be held in LTM (Chao et al., 2022; LaRocque et al., 2015).

Furthermore, these findings are critical to not only reveal how refreshing functions, but further coupled with the previous discussion about how and when LTM influences WM overall, they speak to the theoretical distinctions between the memory systems. As explained previously, embedded processes models (Cowan, 1999; Oberauer, 2002) view WM as an active subset of LTM, and thus WM functions, like refreshing, may operate more effectively when the information is also available in LTM. Accordingly, embedded processes models would predict a Shape × Cue interaction, such that retro-cues are more effective for concrete versus abstract shapes. Conversely, dual-store models (Baddeley, 2000; Barrouillet & Camos, 2015) view WM and LTM as distinct memory systems, and thus LTM should only impact WM overall without moderating its functions. Dual-store models thus predict that no Shape × Cue interaction should be observed. The clear result that retro-cues benefitted WM recall regardless of the shape’s representation in LTM supports the dual-store view. However, future research is necessary to confirm this finding by manipulating other relevant factors to determine its generalizability across different contexts. For example, Matsukura and Vecera (2015) suggested that increasing the number of cued items may diminish the retro-cue effect. Accordingly, perhaps prior knowledge in LTM has no impact on refreshing unless WM capacity is sufficiently exceeded. Such possibilities will be a fruitful area of future work.

To return to the original scenario of following directions to a new restaurant, our results suggest that already being familiar with the city that you are navigating is helpful overall, but thinking back to the last direction from just a moment before is not any worse in a new city. Instead, focusing and switching attention in WM may operate independently from LTM, thereby suggesting that these memory systems are conceptually dissociable.

### Supplementary Information

Below is the link to the electronic supplementary material.

Supplementary file1 (DOCX 755 KB)

## Footnotes

1 Three additional experiments with identical aim and design are reported in the Supplementary Online Materials (SOM). In brief, the primary results of these experiments were consistent with those reported here, but they did not achieve the pre-registered benchmark of an overall effect of shape on calibrated set size; thus, the primary results from the test phase cannot be satisfactorily interpreted. More discussion of the likely methodological reasons for this issue can be found in the SOM.

2 Sampled from the CIELAB color space, with L = 70, a = 20, b = 38, and radius = 60. Experiment 3 had an additional restraint that the distance between the selected colors was at least 360/trial set size to ensure that the colors were not too similar between the shapes of the array, which could cause spurious retrieval errors (e.g., selecting a lure because it was a similar color to the target).

3 One angle along the invisible wheel from 1 to 360 was randomly selected as the first location, and the locations of the rest of the items in the array were determined by adding 360/set size of the trial to each of the next locations in the array based on the location just before it. To make it so that the shapes did not appear evenly spaced and to add more variability akin to the colors of Experiments 1 and 3, a randomly determined amount of “noise” was added to each of the angles of the locations depending on the set size (i.e., between 0 and 162, 108, 72, 32, 24, 12, 6, and 3 for Set Sizes 2, 3, 4, 5, 6, 7, 8, and 9, respectively). These additional angles were determined to ensure that the shapes appeared to be randomly arranged along the invisible wheel but without overlapping.

4 For full transparency, we conducted the experiments as reported here in the following order: S1, S3, pilot, 1, S2, 2, 3. This can be verified by the pre-registration timestamps and explanations within them. For the sake of coherence, we have reorganized their order here.

5 Note that the full 2 (shape: concrete, abstract) × 3 (cue: 0, 1, 2) analysis showed the same pattern of results: a model with two main effects (BF_10_ = 8.40e+28) was strongly preferred to the model including an interaction (BF_main effects/interaction_ = 16.71).

6 Note that although this is widely agreed, there are other explanations for the retro-cue effect besides refreshing (see Souza & Oberauer, 2016, for review).
