# Supplementary material for: What you don’t know can’t hurt you: Retro-cues benefit working memory regardless of prior knowledge in long-term memory
Source: Psychon Bull Rev. 2023 Nov 6;31(3):1–12. doi: 10.3758/s13423-023-02408-w (PMC11192822; doi:10.3758/s13423-023-02408-w)
Supplement: Supplementary file 1 — Supplementary file1 (DOCX 755 KB) [file 13423_2023_2408_MOESM1_ESM.docx]

**Supplementary Online Material**

This supplementary online material has five main sections: (1) a pilot experiment of the concrete/abstract shapes used in the main experiments; (2) three additional pre-registered experiments that were moved from the main text to preserve transparency but also to increase coherence of the main text (originally Experiments 1 to 3, henceforth referred to as Experiments S1 to S3); (3) lure selection analyses of Experiment 3 ; (4) cognitive modeling results of all the experiments that were pre-registered but did not add substantial insights that warranted their inclusion alongside the results reported in the main text; and (5) exploratory analyses of the response times (RTs) of all the experiments requested during the review process of an earlier version of the manuscript. Please refer to the corresponding section for more details.

**Pilot Experiment**

We conducted a “pilot” experiment similar to that of Brady and Störmer’s (2021) Experiment A2 to validate that the stimuli sufficiently manipulate prior knowledge in long-term memory (LTM), thereby removing it as a possible explanation for the lack of an overall effect of shape in the calibration phases of Experiments S1 and S3. Specifically, we presented participants with the original concrete and abstract shapes from Experiments S1 and S3 as well as abstract shapes that were even more strongly scrambled than the abstract shapes from those experiments. Participants were asked to supply a free response to identify what they believed the object to be. If the original concrete and abstract shapes from Experiments S1 and S3 did vary in their representation in LTM, then we expected to find a credible difference in labeling accuracy between the originally used concrete and abstract shapes, albeit the worst accuracy may occur for the more strongly scrambled objects. However, if there was no difference between the original concrete and abstract shapes, then participants may be similarly accurate at identifying the objects, and this would explain the null difference in calibrated set sizes for the objects in Experiments S1 and S3. If this is the case, then we anticipated that we would use the more strongly scrambled abstract shapes for future experiments.

**Method**

***Participants***

Following Brady and Störmer (2021), we aimed to recruit 20 unique participants from the university subject pool (“Sona”; see Table S1). We applied the same inclusion and exclusion criteria as the previous experiments, and no participants from the previous experiments were allowed to participate.

**Table S1**

*Sample Details.*

|  |  | Experiment | | | |
| --- | --- | --- | --- | --- | --- |
| Sample details | | Pilot | S1 | S2 | S3 |
| Total N attempted | | 46 | 66 | 61 | 36 |
| N failed to pass the color blindness/demographic screening phase | | 10 | 14 | 14 | 6 |
| N excluded for pre-registered reasons: | | 14 | 12 | 7 | 10 |
|  | 1. Did not start the experiment after passing the screening phase | 9 | 7 | 1 | 3 |
|  | 2. Incomplete data (e.g., from exiting the full screen, quitting/restarting) | 5 | 5 | 5 | 7 |
|  | 3. Reported issues (e.g., technical) affecting performance | 0 | 0 | 0 | 0 |
|  | 4. Reported not completing experiment in one distraction-free sitting | 0 | 0 | 1 | 0 |
|  | 5. Recall responses exceeded 5s on more than 10% of trials | 0 | 0 | 0 | 0 |
| Final N for analysis after pre-registered exclusions | | 22 | 40 | 40 | 20 |

***Materials and Procedure***

We used the same concrete and abstract shapes from Experiments S1 and S3, as well as additional abstract shapes that are more strongly scrambled using the same diffeomorphic transformation method (Brady & Störmer, 2021; Stojanoski & Cusack, 2014). Figure S1 shows an example of the three types of shapes. Similar to Brady and Störmer, participants viewed shapes sequentially presented for 1 s, followed by a screen that prompted them to type a free response at their own pace about what they believed the shape to be. The shape was presented in a random color drawn from the same color wheel characteristics as in Experiments S1 and S3. We emphasized that participants needed to provide a label and not simply state what it looked like (e.g., “wolf”, not “green”). Each participant completed 120 trials: 40 of each of the original concrete and abstract shapes, and 40 of the more strongly scrambled abstract shapes. These were drawn at random from the full set without replacement (i.e., participants did not label the same object in more than one of the shape conditions).

**Figure S1**

*Example Stimuli from the Pilot Experiment.*


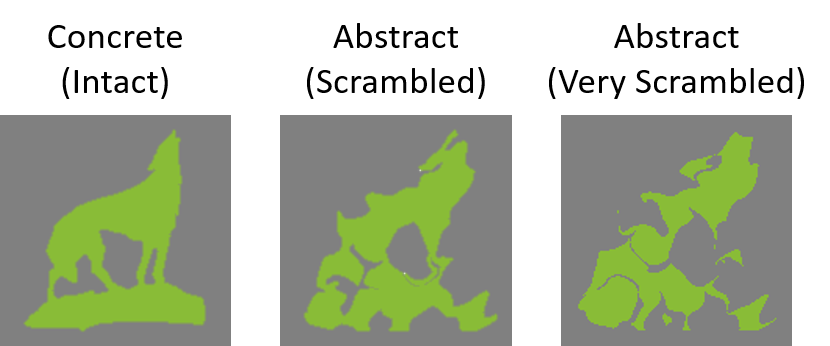


Two raters (co-authors HWC and DTGM) then graded the results manually and completely blind to the shape condition. The raters judged the labels leniently as long as they provided an accurate, object-based description (e.g., “animal” or “dog” was accepted for “wolf”). Inter-rater reliability was very high (95%), and disagreements were resolved with discussion.

**Results and Discussion**

We conducted a one-way repeated measures Bayesian analysis of variance (BANOVA) to assess whether there was evidence for an effect of shape condition on labeling accuracy and RTs, with follow-up Bayesian t-tests to assess the difference between each combination of the shape conditions.

As shown in Figure S2, the results of the pilot experiment confirmed that the nature of the shapes sufficiently varied on the basis of prior knowledge in LTM: The intact, concrete shapes were more likely to be accurately and quickly identified than both scrambled (accuracy: BF_10_ = 3.42e+19; RTs: BF_10_ = 4.29) and very scrambled (accuracy: BF_10_ = 2.19e+20; RTs: BF_10_ = 2.92) abstract shapes. There was some evidence that scrambled shapes were more likely to be accurately identified compared to very scrambled shapes (BF_10_ = 5.67), but there was evidence against a difference in RTs (BF_10_ = 0.32). Thus, the results of the Experiments S1 and S3 are unlikely to be attributable to an insufficient manipulation of prior knowledge in LTM. However, to err on the side of caution, we opted to use the very scrambled abstract shapes for the remaining experiments (i.e., Experiments S2, 1, 2, and 3).

**Figure S2**

*Boxplots of Proportion Accuracy and Response Times (RTs) as a Function of Type of Shape.*


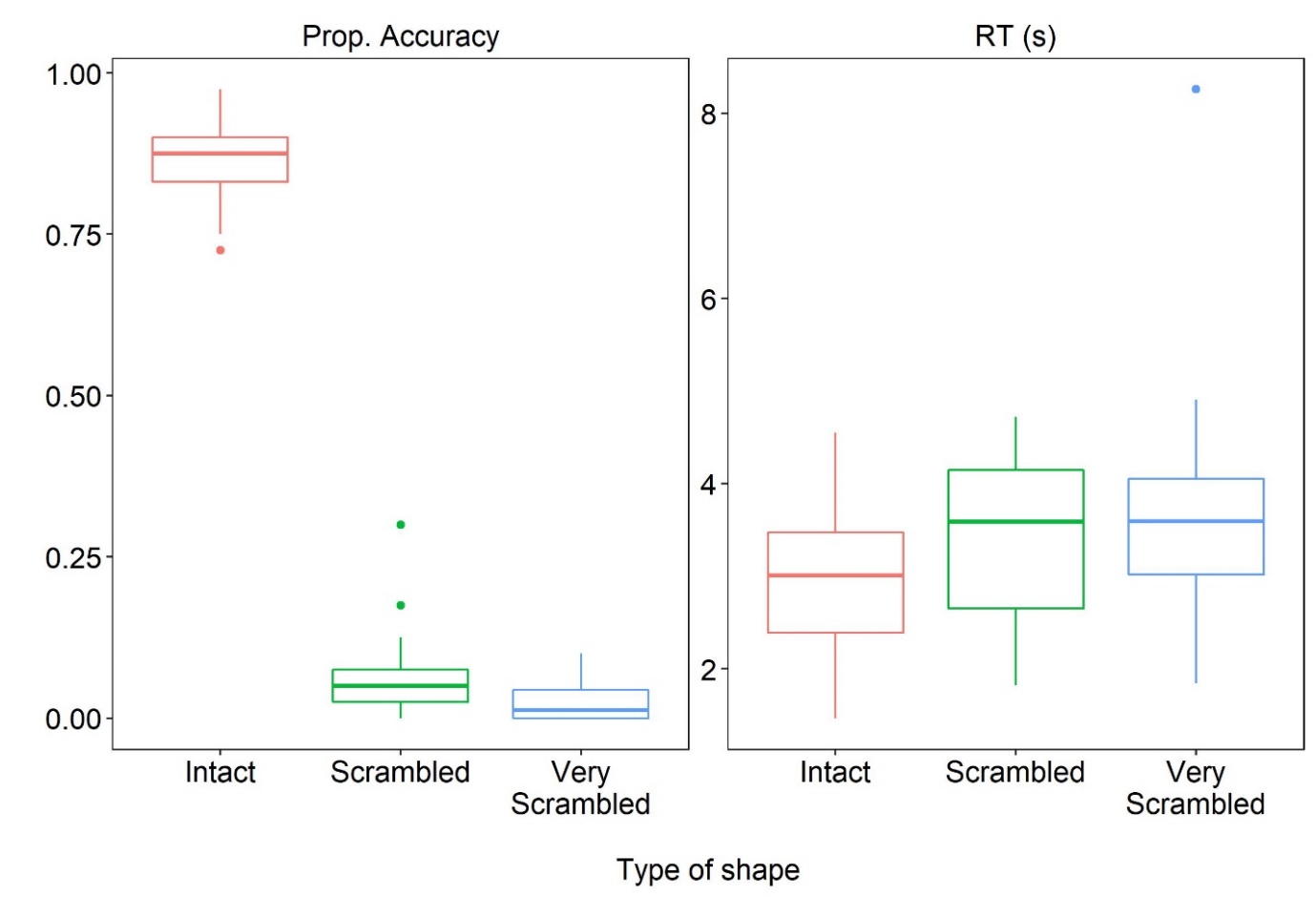


**Experiments S1 to S3**

These three experiments were originally reported in the main text of the manuscript but now reported here in response to reviewer feedback that it was not clear why all five of the original experiments were necessary and to reduce the confusion between experiments in the main text. All three supplementary experiments were very similar to Experiment 1 reported in the main text; the differences between them are summarized in Table S2. To summarize, the main result of interest in these supplementary experiments is consistent with that of the experiments in the manuscript: There was no evidence for a shape x cue interaction, but the null interaction is impossible to interpret because we did not achieve the pre-registered benchmark of a difference in set size between the concrete and abstract shapes.

**Table S2**

*Summary of the Methodological Differences Between Experiments S1 to S3 and the Main Results Speaking to the Pre-Registered Predictions.*

|  | Methodological differences  between experiments | | | Key evidence (BFs) for the pre-registered predictions | | |
| --- | --- | --- | --- | --- | --- | --- |
|  |  |  |  | Calibration phase | | Test phase |
| Exp. | Probe location | Set size range | Level of shape abstractness* | concrete > abstract set size | concrete ≈ abstract recall error | evidence for shape x cue interaction |
| S1 | Original | 3-9 | Scrambled | 0.11 | 4.13 | 1.30 |
| S2 | Original | 2-9 | Very scrambled | 0.30 | 2.34 | 0.23 |
| S3 | Center | 3-9 | Scrambled | 0.61 | 3.96 | 0.31 |
| *Note*. Exp. = experiment; BFs = Bayes factors. * See the Pilot experiment for more information. | | | | | | |

As in Experiment 1, participants viewed colored concrete or abstract shapes, and after a retention interval, they were presented with one of the shapes in dark grey in either its original location (Experiments S1 and S2) or at the center of the screen (Experiment S3) to probe them to recall its color by clicking along the continuous reproduction wheel.^[[1]](#footnote-1)^ Compared to Experiments S1 and S3, Experiment S2 used a more strongly scrambled diffeomorphic transformation that was used in the subsequent experiments reported in the main text of the manuscript. During the calibration block, there was a minimum of 2 (Experiment S2) or 3 (Experiments S1 and S3) shapes. The rest of the methodological and analytic details are the same as reported for Experiment 1 in the main text.

**Results and Discussion**

***Calibration phase: Does prior knowledge in LTM enhance WM overall?***

Although recall error was similar between the two types of shapes during the calibration phase, Experiments S1 to S3 showed evidence *against* the directional hypothesis that the calibrated set size of concrete shapes would be greater than that of abstract shapes (BFs_10_ < 0.61; see Table S2 and Figure S3). This was quite surprising given the literature suggesting that prior knowledge in LTM enhances WM overall (e.g., Engle et al., 1990; Loaiza et al., 2015). Most importantly, it was critical to establish this finding upfront to clearly interpret the next results of the principal hypothesis regarding whether LTM facilitates refreshing in WM. For example, if the next results indicated that there was no interaction between prior knowledge of the shape and the retro-cue effect, then it would be impossible to determine whether the retro-cue benefit was observed in both shape conditions because there truly is no impact of LTM on refreshing or simply because the LTM manipulation was not strong enough (e.g., perhaps the abstract shapes were still identifiable). As the previously presented pilot experiment showed, however, the latter explanation is unlikely.

**Figure S3**

*Set Size and Recall Error as a Function of Shape for Experiments S1 to S3. Large Dark Points Indicate Overall Condition Means, Small Faded Points Indicate Individual Means, and Error Bars Reflect 95% Within-Subjects Confidence Intervals.*


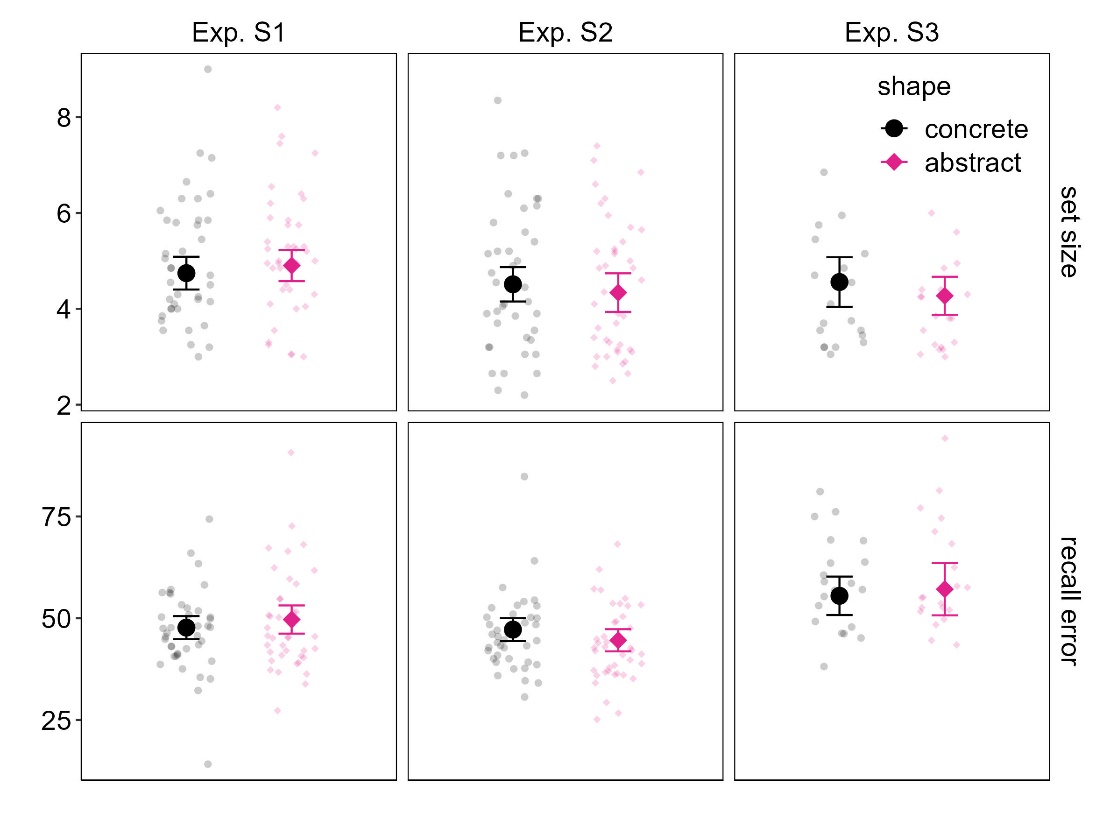


Further reasons for the lack of LTM effect on calibrated set size could also include the possibility that capturing the difference between the concrete and abstract shapes was not possible with a minimum set size of 3 (Experiments S1 and S3). The calibrated recall error in Experiment S3 was particularly high compared to the aim of 40° and to the other experiments, and over half the participants achieved calibrated set sizes of 3.5 or less. Thus, a restricted set size range may have reduced the possibility to observe a difference between the concrete and abstract shapes. Finally, it may be that testing the shapes in their original locations reduced the need for participants to rely on the shapes and their representation in LTM to guide their recall (Experiments S1 and S2). That is, perhaps the participants could effectively ignore the shapes and use the locations of the probes to recall the colors, thereby resulting in a null difference in the calibrated set size to achieve similar recall error between the concrete and abstract shapes. The experiments reported in the main text confirmed many of these suspicions: Reducing the minimum set size to 2 instead of 3 and presenting probes at the center of the screen in the experiments reported in the main text yielded very strong evidence for the predicted difference between concrete and abstract shapes.

**Table S3**

*Results of the Bayesian Analysis of Variance (BANOVA) for Experiments S1 to S3.*

|  |  | Fixed effects (including random effect of participant) | | | |
| --- | --- | --- | --- | --- | --- |
| Exp. | Model (M) ratio | Shape | Cue | Shape + Cue | Shape x Cue |
| S1 | BF_10_ | 0.46 | **123.77** | 63.95 | 83.25 |
|  | Best M/M | 268.83 | Best | 1.94 | 1.49 |
| S2 | BF_10_ | 0.17 | **19.95** | 3.51 | 0.82 |
|  | Best M/M | 113.69 | Best | 5.69 | 24.47 |
| S3 | BF_10_ | 0.23 | **1584.93** | 366.77 | 113.66 |
|  | Best M/M | 6854.84 | Best | 4.32 | 13.94 |
| *Note.* Each reported Bayes factor (BF) refers to the evidence for the alternative model (BF_10_) for each effect (shown in the different columns) relative to the null model (i.e., intercept-only model). The best model is shown in boldface in the first row for each experiment, and the second row for each experiment compares the best model in the numerator to each of the other models in the denominator. | | | | | |
|  |  |  |  |  |  |
|  |  |  |  |  |  |
|  |  |  |  |  |  |

***Test phase: Does prior knowledge in LTM facilitate refreshing in WM?***

Second and most importantly, we assessed the evidence for our principal hypothesis regarding whether LTM facilitates refreshing in WM by using a 2 (shape) x 2 (cue) repeated measures BANOVA on recall error during the test blocks. The results are summarized in Table S3 and Figure S4. Table S2 also summarizes the BFs that show the unique evidence for an interaction (i.e., BF_interaction/main effects_; Wetzels et al., 2012). In all three experiments the best model included only a retro-cue effect: In Experiment S1, the cue-only model was only weakly preferred to the next best model that included the shape x cue interaction (BF_cue/interaction_ = 1.49). Experiments S2 and S3 showed much more substantial evidence in favor of only a retro-cue effect over and above the evidence for the model including an interaction (BFs_cue/interaction_ > 13.94). However, as explained previously, the lack of an overall effect of shape in the calibration phases of these experiments rendered these results difficult to interpret. The experiments reported in the main text of the manuscript achieved this benchmark, and therefore we do not strongly interpret the results of Experiments S1 to S3 here.

**Figure S4**

*Recall Error as a Function of Shape and Cue for Experiments S1 to S3. Large Dark Lines Indicate Overall Condition Means, Faded Lines Indicate Individual Means, and Error Bars Reflect 95% Within-Subjects Confidence Intervals.*


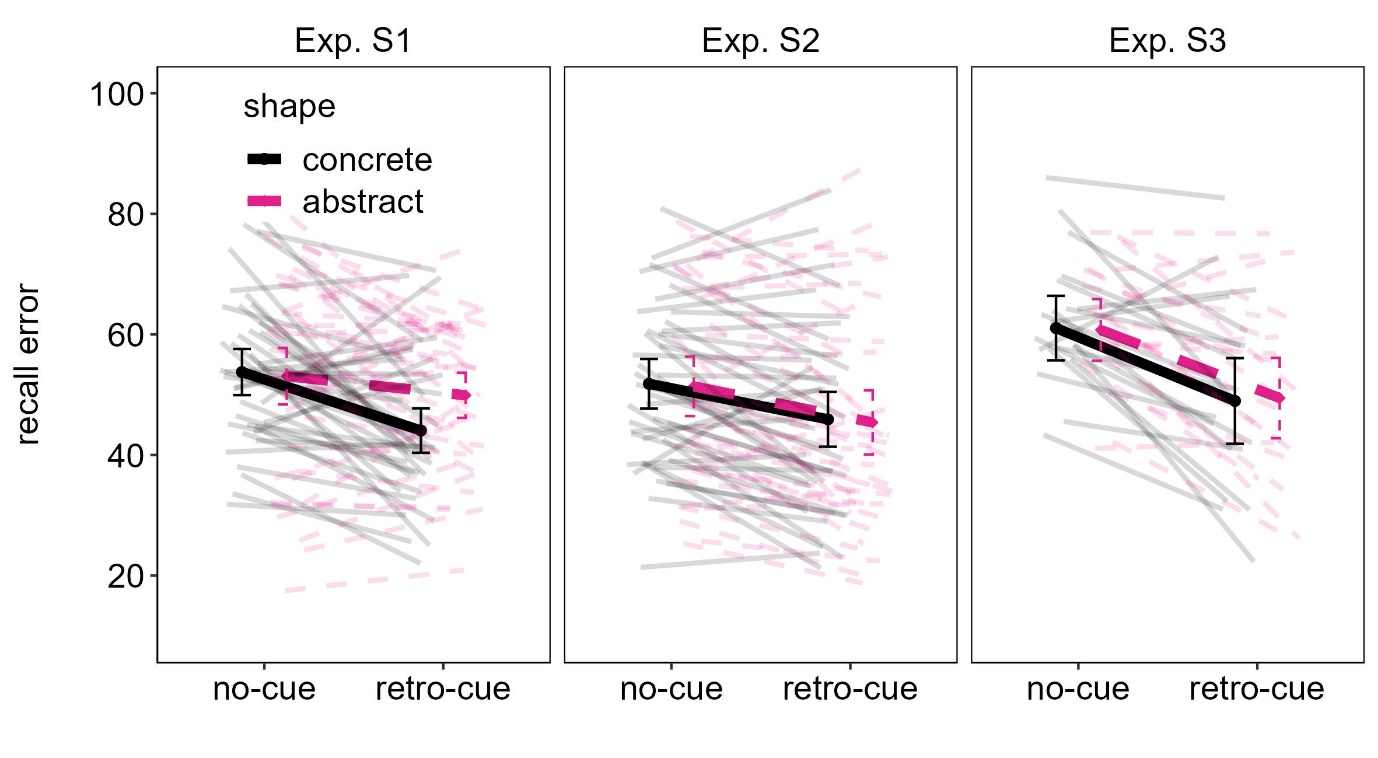


***Summary and conclusions***

These results suggest that when participants could rely on the location of the probes (Experiments S1 and S2) or when the set size range was restricted (Experiment S3), the representation of the information in LTM was less useful to WM, thus resulting in a null effect of shape on the calibrated set sizes of these experiments. This may suggest that the benefit of prior knowledge is not necessarily fixed and universal. That is, if information from LTM is not necessary or useful to the WM task (e.g., when a probe’s location is available to guide recall; Experiments S1 and S2), then an overall effect of LTM may not be observed. This is in line with recent evidence that WM only recruits information from LTM when it is beneficial to do so (Bartsch & Shepherdson, 2021; Mızrak & Oberauer, 2022; Oberauer, Awh, et al., 2017; Sobrinho & Souza, 2023). Thus, the current results refine a previously uncontroversial assumption that LTM regularly impacts WM overall, suggesting instead that its overall effect is conditional on whether the information from LTM is beneficial to WM. If WM can function without relying on LTM, then an overall effect of LTM may not be observed.

**Experiment 3 Lure Selection Analysis Results**

This section concerns the results of the two additional analyses that considered differences in lure selection according to whether the lures were spatial neighbors (for all cue conditions) or previously cued (for the double retro-cue condition) of concrete and abstract shapes from Experiment 3 in the main text. The descriptive statistics are shown in Table S4. Note that these cells were unavoidably uneven in favor of a lure being a neighbor or previously cued given that set size was individually calibrated. For example, for trials where set size was 2, then the lure was necessarily a spatial neighbor and previously cued. So, the reader should bear this imbalance in mind when interpreting the next results.

**Table S4**

*Means (and Standard Deviations) of the Proportion of Selected Lures (Spatial Neighbors or Previously Cued) as a Function of Cue and Shape Conditions in Experiment 3.*

|  | no-cue | | 1 cue (focus) | | 2 cues (switch) | |
| --- | --- | --- | --- | --- | --- | --- |
| lure type | concrete | abstract | concrete | abstract | concrete | abstract |
| neighbor | 0.28 (0.09) | 0.25 (0.07) | 0.16 (0.09) | 0.15 (0.09) | 0.16 (0.11) | 0.16 (0.07) |
| non-neighbor | 0.24 (0.14) | 0.29 (0.13) | 0.17 (0.17) | 0.17 (0.11) | 0.15 (0.13) | 0.20 (0.16) |
|  |  |  |  |  |  |  |
| previously cued | - | - | - | - | 0.17 (0.13) | 0.20 (0.15) |
| not previously cued | - | - | - | - | 0.16 (0.17) | 0.22 (0.14) |

A 2 (cue) x 2 (shape) x 2 (lure type) within-subjects BANOVA on the selection of spatial neighbors showed an overall main effect of cue (BF_10_ = 2.03e+12) that was substantially preferred (BF = 4.81) to the next best model that also included a main effect of shape (BF_10_ = 4.23e+11). As is evident in Table S4, participants selected lures most often during no-cue trials relative to single and double retro-cue trials, regardless of the nature of the shape or whether the lure was a spatial neighbor. Furthermore, a 2 (shape) x 2 (lure type) within-subjects BANOVA on the selection of previously cued lures during the double retro-cue trials showed an overall effect of shape (BF_10_ = 80.65) that was substantially preferred (BF = 6.01) to the next best model that included a main effect of lure type (BF_10_ = 13.43). As is evident in Table S4, lures were selected most often during the abstract versus concrete block overall, regardless of whether the lure was previously cued or not. Overall, these results suggest that whether the lure was a spatial neighbor or previously cued did not impact the pattern of lure selection.

**Cognitive Modeling Results**

**Three-Parameter Mixture Model Results of Experiments S1 to Experiment 2**

According to our pre-registrations, we fit recall error from Experiments S1 to Experiment 2 with a hierarchical Bayesian three-parameter mixture model (Oberauer, Stoneking, et al., 2017). This mixture model assumes that observed recall error reflects the contributions of (1) the probability that the tested shape’s color is in WM with a (2) specific precision, and the probabilities of (3) binding errors (recalling the color of another shape) or (4) guessing when the information is not in WM. The full model and its priors can be found on the OSF. The model was fit using rjags (Plummer, 2016) via Markov Chain Monte Carlo (MCMC) sampling. We checked for convergence of the four MCMC chains via visual inspection as well as verified that the R-hat statistic was close to 1 for all parameters. We also conducted posterior predictive checks to ensure appropriate model fit to the data. We pre-registered this analysis of observed recall error by examining the evidence for these retro-cue benefits in the underlying cognitive parameters as estimated from the mixture model. We expected the results to cohere with those of recall error, such that the retro-cue benefit may be particularly evident in the probability of recalling the target color (Arnicane & Souza, 2021), and its strength may depend on whether the color belonged to a concrete or abstract shape. Table S5 summarizes the parameter estimate results across the experiments. Plots of the parameter estimates for each experiment can be found on the OSF.

The results showed credible retro-cue effects in the probability of recalling the target in both shape conditions in Experiments S3 and 1. In Experiment S1, there was a credible retro-cue effect in the concrete condition but not the abstract condition, in line with the recall error results of this experiment suggesting a shape x cue interaction. In Experiment S2, there were no credible effects of condition for any of the parameters. The retro-cue effect in observed performance was weakest in this experiment compared to the others, likely due to the issues discussed in the main text. In Experiment 2, there was a credible retro-cue effect in the abstract condition but not in the concrete condition. It is not clear why this pattern

**Table S5**

*Summary of Mean Parameter Estimates [and 95% Highest-Density Intervals]*

| Exp. | Shape | Cue | *P*(Target) | Precision | *P*(Binding error) | *P*(Guessing) |
| --- | --- | --- | --- | --- | --- | --- |
| S1 | Concrete | No-cue | 0.51 [0.45, 0.57] | 5.24 [4.27, 6.29] | 0.22 [0.16, 0.28] | 0.27 [0.20, 0.34] |
|  | Concrete | Retro-cue | 0.64 [0.58, 0.69] | 5.92 [5.03, 6.91] | 0.23 [0.18, 0.28] | 0.13 [0.08, 0.18] |
|  | *Concrete retro-cue effect* | | **0.13 [0.05, 0.21]** | 0.68 [-0.79, 2.06] | 0.01 [-0.07, 0.09] | **-0.14 [-0.22, -0.05]** |
|  |  |  |  |  |  |  |
|  | Abstract | No-cue | 0.51 [0.44, 0.58] | 5.79 [4.68, 7.03] | 0.24 [0.18, 0.31] | 0.25 [0.18, 0.32] |
|  | Abstract | Retro-cue | 0.54 [0.49, 0.60] | 5.94 [5.01, 6.93] | 0.28 [0.23, 0.34] | 0.17 [0.12, 0.22] |
|  | *Abstract retro-cue effect* | | 0.03 [-0.05, 0.12] | 0.15 [-1.39, 1.67] | 0.04 [-0.04, 0.12] | -0.07 [-0.16, 0.01] |
|  |  |  |  |  |  |  |
| S2 | Concrete | No-cue | 0.57 [0.51, 0.63] | 5.16 [4.01, 6.32] | 0.19 [0.14, 0.25] | 0.24 [0.18, 0.30] |
|  | Concrete | Retro-cue | 0.64 [0.57, 0.72] | 6.79 [5.43, 8.22] | 0.13 [0.06, 0.21] | 0.22 [0.16, 0.29] |
|  | *Concrete retro-cue effect* | | 0.07 [-0.02, 0.17] | 1.63 [-0.17, 3.49] | -0.06 [-0.15, 0.03] | -0.01 [-0.10, 0.07] |
|  |  |  |  |  |  |  |
|  | Abstract | No-cue | 0.55 [0.48, 0.61] | 5.83 [4.90, 6.75] | 0.18 [0.13, 0.23] | 0.28 [0.21, 0.35] |
|  | Abstract | Retro-cue | 0.63 [0.55, 0.70] | 6.46 [5.34, 7.63] | 0.19 [0.13, 0.25] | 0.18 [0.11, 0.26] |
|  | *Abstract retro-cue effect* | | 0.08 [-0.03, 0.18] | 0.64 [-0.83, 2.13] | 0.01 [-0.06, 0.10] | -0.09 [-0.20, 0.01] |
|  |  |  |  |  |  |  |
| S3 | Concrete | No-cue | 0.41 [0.33, 0.48] | 6.39 [4.28, 8.71] | 0.20 [0.12, 0.29] | 0.39 [0.28, 0.50] |
|  | Concrete | Retro-cue | 0.54 [0.45, 0.63] | 8.10 [5.76, 10.77] | 0.14 [0.06, 0.22] | 0.31 [0.21, 0.42] |
|  | *Concrete retro-cue effect* | | **0.14 [0.02, 0.26]** | 1.71 [-1.76, 5.09] | -0.06 [-0.18, 0.06] | -0.08 [-0.23, 0.08] |
|  |  |  |  |  |  |  |
|  | Abstract | No-cue | 0.39 [0.32, 0.46] | 5.64 [3.63, 7.56] | 0.44 [0.36, 0.53] | 0.17 [0.08, 0.26] |
|  | Abstract | Retro-cue | 0.53 [0.44, 0.61] | 7.67 [5.91, 9.73] | 0.33 [0.25, 0.41] | 0.14 [0.04, 0.24] |
|  | *Abstract retro-cue effect* | | **0.14 [0.03, 0.25]** | 2.03 [-0.79, 4.89] | -0.11 [-0.23, 0.01] | -0.03 [-0.16, 0.11] |
|  |  |  |  |  |  |  |
| 1 | Concrete | No-cue | 0.47 [0.40, 0.54] | 7.46 [5.57, 9.47] | 0.27 [0.19, 0.34] | 0.26 [0.17, 0.35] |
|  | Concrete | Retro-cue | 0.64 [0.56, 0.72] | 9.08 [7.18, 11.01] | 0.18 [0.11, 0.26] | 0.18 [0.11, 0.26] |
|  | *Concrete retro-cue effect* | | **0.17 [0.07, 0.28]** | 1.61 [-1.24, 4.39] | -0.09 [-0.19, 0.01] | -0.08 [-0.20, 0.04] |
|  |  |  |  |  |  |  |
|  | Abstract | No-cue | 0.52 [0.44, 0.60] | 7.42 [5.35, 9.72] | 0.26 [0.17, 0.35] | 0.22 [0.14, 0.30] |
|  | Abstract | Retro-cue | 0.64 [0.57, 0.71] | 11.93 [9.48, 14.42] | 0.20 [0.14, 0.27] | 0.16 [0.09, 0.23] |
|  | *Abstract retro-cue effect* | | **0.12 [0.01, 0.23]** | **4.51 [1.16, 7.79]** | -0.06 [-0.17, 0.06] | -0.07 [-0.17, 0.04] |
|  |  |  |  |  |  |  |
| 2 | Concrete | No-cue | 0.59 [0.53, 0.65] | 16.54 [12.46, 20.82] | 0.16 [0.12, 0.21] | 0.24 [0.17, 0.32] |
|  | Concrete | Retro-cue | 0.66 [0.59, 0.72] | 22.02 [18.11, 26.16] | 0.11 [0.07, 0.14] | 0.24 [0.16, 0.31] |
|  | *Concrete retro-cue effect* | | 0.06 [-0.02, 0.15] | 5.49 [-0.45, 11.20] | -0.06 [-0.11, 0.00] | -0.01 [-0.11, 0.10] |
|  |  |  |  |  |  |  |
|  | Abstract | No-cue | 0.52 [0.46, 0.57] | 26.16 [21.55, 31.25] | 0.08 [0.05, 0.11] | 0.41 [0.35, 0.46] |
|  | Abstract | Retro-cue | 0.60 [0.55, 0.66] | 27.50 [23.19, 32.06] | 0.10 [0.07, 0.12] | 0.30 [0.24, 0.36] |
|  | *Abstract retro-cue effect* | | **0.09 [0.01, 0.16]** | 1.34 [-5.19, 7.98] | 0.02 [-0.02, 0.06] | **-0.10 [-0.19, -0.02]** |
| *Note.* Retro-cue effects in boldface font indicate credible effects. | | | | | | |

occurred due to a credible retro-cue effect in both conditions in observed performance. The remaining credible effects in the other parameters were less systematic across experiments. Overall, these results are in line with other similar research that retro-cues tend to benefit observed performance due to an increased likelihood of recalling the target item compared to the no-cue baseline. Importantly, this largely occurred for both concrete and abstract shapes, in line with the conclusions in the main text.

**Multinomial Processing Tree Model Results of Experiment 3**

We fit a hierarchical Bayesian multinomial processing tree (MPT) model to estimate the contributions of binding and item memory to the recognition decisions of Experiment 3. As a reminder, Experiment 3 followed a three-alternative forced choice procedure wherein participants selected one option among the correct target, a lure presented during the trial but not in the correct color, and a new option that was not presented in the trial. This model was very similar to prior work (e.g., Bartsch et al., 2019; Loaiza & Srokova, 2020) that assumes that correctly recalling the target can occur due to accurate binding memory, but in absence of binding memory, participants may guess with equal probability between the target and the lure on the basis of item memory. In the absence of item memory, participants guess with equal probability between all the options. The model was fit using the R package TreeBUGS (Heck et al., 2018), all of the information of which can be found on the OSF. As was the case for the mixture modeling, we ensured adequate convergence of the MCMC chains and fit of the model to the data. The aim of the analysis was to determine whether distinguishing between binding and item memory added insight into the pattern of the observed performance results reported in the main text. To draw inferences, we inspected and report the mean differences [and 95% credibility intervals] of the parameter estimates between the retro-cue conditions for each type of shape to determine whether the single (one-cue) and double (two-cues) retro-cue benefits were similar between concrete and abstract shapes.

**Figure S5**

*Posterior Parameter Estimates of Binding Memory and Item Memory as a Function of Shape and Cue for Experiment 3. Large Dark Lines Indicate Overall Means, Faded Lines Indicate Individual Means, and Error Bars Reflect 95% Credibility Intervals.*


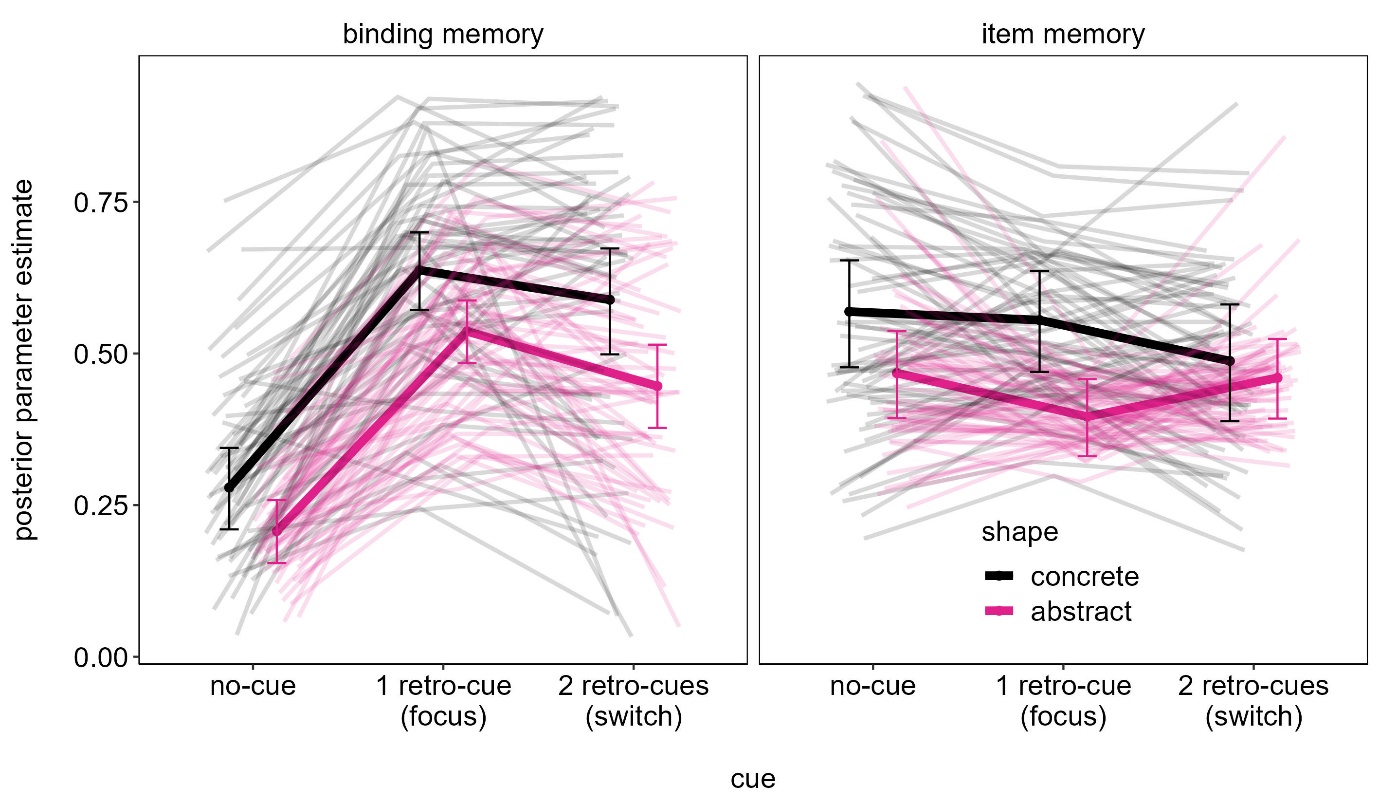


As can be seen in Figure S5, the binding memory parameter estimates followed the pattern of observed performance results reported in the main text: Relative to the no-cue baseline, both single and double retro-cues benefitted binding memory of the concrete shapes (single = 0.36 [0.28, 0.44]; double = 0.31 [0.22, 0.40]), with no credible difference in binding memory between the single and double retro-cues (0.05 [-0.03, 0.13]). Binding memory of abstract shapes also benefitted from both single (0.33 [0.26, 0.40]) and double (0.24 [0.16, 0.31]) retro-cues, but unlike concrete shapes there was a credible difference between them (0.09 [0.02, 0.16]). Further research will be required to determine whether this is a reliable difference. Finally, item memory showed a very different pattern, such that there were no credible retro-cue effects for either concrete or abstract shapes. Overall, these results suggest that the retro-cue effects observed in Experiment 3 were specific to binding memory. Furthermore, the general pattern of results coheres with the main conclusion from the task performance results that both focusing (single retro-cues) and switching (double retro-cues) attention function similarly regardless of prior knowledge in LTM.

**Response Times Results of All Experiments**

Following a reviewer’s suggestion, we also conducted an exploratory analysis of the retrieval decision RTs to examine the evidence for a shape x cue interaction. Across the experiments, there was a clear retro-cue effect (see Table S6), such that RTs were faster overall during retro-cue compared to no-cue trials, replicating a great deal of prior work (see Souza & Oberauer, 2016 for review).

**Table S6**

*Means (and Standard Deviations) of Response Times (RTs, s) as a Function of Experiment, Shape, and Cue Condition.*

| Exp. | Shape | No-Cue | 1 Retro-Cue | 2 Retro-Cues |
| --- | --- | --- | --- | --- |
| S1 | Concrete | 1.91 (0.36) | 1.62 (0.38) | - |
|  | Abstract | 1.90 (0.37) | 1.67 (0.37) | - |
|  |  |  |  |  |
| S2 | Concrete | 1.81 (0.42) | 1.53 (0.37) | - |
|  | Abstract | 1.83 (0.42) | 1.59 (0.35) | - |
|  |  |  |  |  |
| S3 | Concrete | 1.89 (0.29) | 1.51 (0.21) | - |
|  | Abstract | 1.78 (0.34) | 1.45 (0.23) | - |
|  |  |  |  |  |
| 1 | Concrete | 1.84 (0.34) | 1.35 (0.29) | - |
|  | Abstract | 1.85 (0.43) | 1.48 (0.33) | - |
|  |  |  |  |  |
| 2 | Concrete | 1.46 (0.37) | 0.92 (0.28) | - |
|  | Abstract | 1.48 (0.29) | 1.00 (0.28) | - |
|  |  |  |  |  |
| 3 | Concrete | 1.57 (0.38) | 1.30 (0.32) | 1.28 (0.31) |
|  | Abstract | 1.51 (0.39) | 1.36 (0.33) | 1.32 (0.31) |

Table S7 further shows that the model including only a retro-cue effect was either weakly (Experiment 1) or substantially (Experiments S1, S2, and 3) preferred to the full model including an interaction between shape and cue. For Experiments S3 and 2, the best model included a main effect of shape, but in these instances the best model was weakly preferred to the simpler models including only a retro-cue effect. Indeed, follow-up analyses showed substantial evidence *against* a difference between the concrete and abstract RTs for each of the cue conditions in Experiments S3 and 1 (BF_01_s > 3.37). For Experiments 2 and 3, there was weak to strong evidence (BF_10_s ranging between 2.74-156) for a difference between concrete and abstract RTs, but importantly, this was consistent across cue conditions. That is, even when RTs were slower during abstract than concrete trials, this was often the case regardless of cue condition (except Experiment 3 where concrete RTs were slower during the no-cue trials). Thus, the exploratory RT analysis shows generally consistent conclusions with the accuracy analyses reported in the main text: Retro-cues decreased RTs regardless of the nature of the shape.

**Table S7**

*Results of the Bayesian Analysis of Variance (BANOVA) on RTs for Each Experiment.*

|  |  | Fixed effects (including random effect of participant) | | | |
| --- | --- | --- | --- | --- | --- |
| Exp. | Model (M) ratio | Shape | Cue | Shape + Cue | Shape x Cue |
| S1 | BF_10_ | 0.21 | **1.33E+13** | 3.21E+12 | 1.47E+12 |
|  | Best M/M | 6.41E+13 | Best | 4.14 | 9.05 |
| S2 | BF_10_ | 0.26 | **1.17E+11** | 4.06E+10 | 1.10E+10 |
|  | Best M/M | 4.42E+11 | Best | 2.88 | 10.64 |
| S3 | BF_10_ | 0.58 | 6.43E+08 | **1.05E+09** | 3.78E+08 |
|  | Best M/M | 1.81E+09 | 1.63 | Best | 2.78 |
| 1 | BF_10_ | 0.36 | **1.94E+12** | 1.58E+12 | 1.12E+12 |
|  | Best M/M | 3.13E+12 | Best | 1.23 | 1.74 |
| 2 | BF_10_ | 0.25 | 2.67E+38 | **2.88E+38** | 1.80E+38 |
|  | Best M/M | 1.15E+39 | 1.08 | Best | 1.61 |
| 3 | BF_10_ | 0.13 | **1.60E+14** | 2.18E+13 | 1.58E+13 |
|  | Best M/M | 1.24E+15 | Best | 7.40 | 10.20 |
| *Note.* Each reported Bayes factor (BF) refers to the evidence for the alternative model (BF_10_) for each effect (shown in the different columns) relative to the null model (i.e., intercept-only model). The best model is shown in boldface in the first row for each experiment, and the second row for each experiment compares the best model in the numerator to each of the other models in the denominator. | | | | | |
|  |  |  |  |  |  |
|  |  |  |  |  |  |
|  |  |  |  |  |  |

**References**

Arnicane, A., & Souza, A. S. (2021). Assessing the robustness of feature-based selection in visual working memory. *Journal of Experimental Psychology: Human Perception and Performance*, *47*(5), 731–758. https://doi.org/10.1037/xhp0000911

Bartsch, L. M., Loaiza, V. M., & Oberauer, K. (2019). Does limited working memory capacity underlie age differences in associative long-term memory? *Psychology and Aging*, *34*, 282–293. https://doi.org/10.1037/pag0000317

Bartsch, L. M., & Shepherdson, P. (2021). Freeing capacity in working memory (WM) through the use of long-term memory (LTM) representations. *Journal of Experimental Psychology: Learning, Memory, and Cognition*. https://doi.org/10.1037/xlm0001024

Brady, T. F., & Störmer, V. S. (2021). The role of meaning in visual working memory: Real-world objects, but not simple features, benefit from deeper processing. *Journal of Experimental Psychology: Learning, Memory, and Cognition*, No Pagination Specified-No Pagination Specified. https://doi.org/10.1037/xlm0001014

Engle, R. W., Nations, J. K., & Cantor, J. (1990). Is ‘working memory capacity’ just another name for word knowledge? *Journal of Educational Psychology*, *82*(4), 799–804. https://doi.org/10.1037/0022-0663.82.4.799

Heck, D. W., Arnold, N. R., & Arnold, D. (2018). TreeBUGS: An R package for hierarchical multinomial-processing-tree modeling. *Behavior Research Methods*, *50*(1), 264–284. https://doi.org/10.3758/s13428-017-0869-7

Loaiza, V. M., Duperreault, K. A., Rhodes, M. G., & McCabe, D. P. (2015). Long-term semantic representations moderate the effect of attentional refreshing on episodic memory. *Psychonomic Bulletin & Review*, *22*(1), 274–280. https://doi.org/10.3758/s13423-014-0673-7

Loaiza, V. M., & Srokova, S. (2020). Semantic Relatedness Corrects the Age-Related Binding Deficit in Working Memory and Episodic Memory. *The Journals of Gerontology: Series B*, *75*(9), 1841–1849. https://doi.org/10.1093/geronb/gbz055

Mızrak, E., & Oberauer, K. (2022). Working memory recruits long-term memory when it is beneficial: Evidence from the Hebb effect. *Journal of Experimental Psychology: General*, *151*(4), 763–780. https://doi.org/10.1037/xge0000934

Oberauer, K., Awh, E., & Sutterer, D. W. (2017). The role of long-term memory in a test of visual working memory: Proactive facilitation but no proactive interference. *Journal of Experimental Psychology: Learning, Memory, and Cognition*, *43*(1), 1–22. https://doi.org/10.1037/xlm0000302

Oberauer, K., Stoneking, C., Wabersich, D., & Lin, H.-Y. (2017). Hierarchical Bayesian measurement models for continuous reproduction of visual features from working memory. *Journal of Vision*, *17*(5), 11. https://doi.org/10.1167/17.5.11

Plummer, M. (2016). *rjags: Bayesian graphical models using MCMC* [Computer software]. https://cran.r-project.org/web/packages/rjags/rjags.pdf

Sobrinho, N. D., & Souza, A. S. (2023). The interplay of long-term memory and working memory: When does object-color prior knowledge affect color visual working memory? *Journal of Experimental Psychology: Human Perception and Performance*, *49*(2), 236–262. https://doi.org/10.1037/xhp0001071

Souza, A. S., & Oberauer, K. (2016). In search of the focus of attention in working memory: 13 years of the retro-cue effect. *Attention, Perception, & Psychophysics*, *78*(7), 1839–1860. https://doi.org/10.3758/s13414-016-1108-5

Stojanoski, B., & Cusack, R. (2014). Time to wave good-bye to phase scrambling: Creating controlled scrambled images using diffeomorphic transformations. *Journal of Vision*, *14*(12), 6–6. https://doi.org/10.1167/14.12.6

Wetzels, R., Grasman, R. P. P. P., & Wagenmakers, E.-J. (2012). A Default Bayesian Hypothesis Test for ANOVA Designs. *The American Statistician*, *66*(2), 104–111. https://doi.org/10.1080/00031305.2012.695956

1. Recall error was slightly underestimated by about 3° in Experiments S1 and S3 due to a programming error that was fixed for the other experiments. Note that this error unsystematically affected performance across the trials, and thus the general pattern of results from Experiments S1 and S3 is unlikely to be affected. [↑](#footnote-ref-1)
